# Structural–Material Coupling Enabling Broadband Absorption for a Graphene Aerogel All-Medium Metamaterial Absorber

**DOI:** 10.3390/nano16010018

**Published:** 2025-12-22

**Authors:** Kemeng Yan, Yuhui Ren, Jiaxuan Zhang, Man Song, Xuhui Du, Meijiao Lu, Dingfan Wu, Yiqing Li, Jiangni Yun

**Affiliations:** 1 School of Electronic Information, Northwest University, Xi’an 710127, China; 202333011@stumail.nwu.edu.cn (K.Y.); jiaxuanz@stumail.nwu.edu.cn (J.Z.); meijiaolu@stumail.nwu.edu.cn (M.L.); 202522006@stumail.nwu.edu.cn (D.W.); 202533912@stumail.nwu.edu.cn (Y.L.); niniyun@nwu.edu.cn (J.Y.); 2 Xi’an HengDa Microwave Technology Development Co., Ltd., Xi’an 710100, China; haohaizi0679@163.com (M.S.); xiaoxu-100@126.com (X.D.); 3 Department of Physics, McGill University, Montreal, QC H3A 2T8, Canada

**Keywords:** graphene aerogel, relaxation polarization loss, all-medium metamaterial absorber, electromagnetic wave absorption

## Abstract

All-medium metamaterial absorbers (MMAs) have attracted considerable attention for ultra-broadband electromagnetic wave (EMW) absorption. Herein, a lightweight graphene aerogel (GA) was synthesized through a low-temperature, atmospheric-pressure reduction route. Benefiting from its 3D porous network, enriched oxygen-containing functional groups, and improved graphitization, the GA offers diverse intrinsic attenuation pathways and a limited effective absorption bandwidth (EAB) of only 6.46 GHz (11.54–18.00 GHz at 1.95 mm). To clarify its attenuation mechanism, nonlinear least-squares fitting was used to quantitatively separate electrical loss contributions. Compared with graphene, the GA shows markedly superior attenuation capability, making it a more suitable medium for MMA design. Guided by equivalent circuit modeling, a stacked frustum-configured GA-based MMA (GA-MMA) was developed, where structure-induced resonances compensate for the intrinsic absence of magnetic components in the GA, thereby substantially broadening its absorption range. The GA-MMA achieves an EAB of 40.7 GHz (9.1–49.8 GHz, reflection loss < −10 dB) and maintains stable absorption under incident angles up to ± 70°. Radar cross-section simulations further indicate its potential in electromagnetic interference mitigation, human health protection, and defense information security. This work provides a feasible route for constructing ultralight and broadband MMAs by coupling electrical loss with structural effects.

## 1. Introduction

The increasing utilization of electromagnetic waves (EMWs) for information and energy transmission underscores the critical need for high-performance electromagnetic (EM) absorbing materials. In military applications, EM shielding and stealth technologies significantly enhance the survivability and fighting effectiveness of equipment [1,2,3]. In civilian contexts, the rapid deployment of 5G communication infrastructure and wireless network nodes necessitates effective mitigation of EM interference and potential health hazards [4,5,6]. Thus, developing advanced EM absorbers has become a key research focus [7,8].

Metamaterial absorbers (MAs) are artificially engineered materials that regulate EM wave absorption through periodic subwavelength structures [9,10,11,12,13,14]. Early MA designs mainly relied on the classical metal–dielectric–metal (MDM) sandwich architecture [15,16,17], in which impedance matching allows incident waves to enter the absorber, while localized resonances within each unit-cell dissipate EM energy over limited frequency ranges. Although multi-resonant designs can broaden the absorption band to some extent, the achievable bandwidth is still constrained by the discrete nature of these resonances. Compared with conventional MDM-type MAs, all-medium metamaterial absorbers (MMAs) employ nonmetallic media, such as carbon-based materials, ceramics, or conductive polymers, together with a metallic backplate to suppress transmission [18,19,20]. MMAs are attractive due to their low density and structural adaptability, and their operating frequencies can be tailored from the millimeter-wave to the terahertz regime through geometric design. Yet, in the microwave band, achieving broadband absorption with all-dielectric or all-medium MMAs remains challenging, since broadband impedance matching and sufficient energy dissipation must be realized simultaneously without relying on metal-type surface-current loss channels [21,22,23,24,25]. Consequently, continuous ultra-wideband absorption cannot be achieved by high-Q dielectric resonances alone and instead requires deliberate loss and impedance engineering. In this context, true metamaterial absorbers rely on artificially designed subwavelength unit-cell structures, where structure-induced resonant responses play a central role in achieving impedance matching and enhanced absorption. Although subwavelength resonant structures have been widely studied in optical and integrated metamaterials, their working frequencies, material systems, and loss mechanisms differ substantially from those required for microwave absorption [26,27]. Therefore, it is an effective strategy that couples microscopic loss processes with macroscopic structural resonances in MMAs to develop new metamaterial devices in EMW absorption.

Carbon-based materials, especially graphene and its derivatives, have been widely studied as EM wave absorbents because of their low density, good conductivity, and abundant defects [28,29]. By assembling graphene sheets into a 3D porous framework, graphene aerogels (GAs) combine ultralight weight with adjustable electrical properties and abundant internal interfaces [30,31,32,33]. These features promote multiple reflections of EMWs, while dipole polarization, interfacial polarization, and hopping conduction contribute to electrical loss under alternating fields. In contrast to metamaterial absorbents, conventional porous carbon-based absorbents are usually treated as homogeneous lossy media, and their absorption performance is mainly governed by intrinsic electrical loss mechanisms. Nevertheless, most GA-based EM absorbents, together with other porous carbon absorbents derived from biomass or metal–organic frameworks, mainly rely on electrical loss for EM attenuation. Since carbon materials are nonmagnetic, broadband impedance matching and bandwidth extension are difficult to achieve at high frequencies, and the effective absorption bandwidth (EAB) therefore remains limited, even when porosity engineering, heteroatom doping, or composite modification is applied. Moreover, such absorbents are usually treated as homogeneous lossy media, and their absorption performance is tuned mainly by adjusting conductivity or filler content, which provides limited control over resonance behavior and restricts further broadband improvement.

Herein, an ultralight graphene aerogel-based all-medium metamaterial absorber (GA-MMA) is proposed. In this design, graphene aerogel is employed as a lossy medium within a tailored metamaterial unit-cell. To obtain quantitative insight into its intrinsic attenuation behavior, the electrical loss of the graphene aerogel is analyzed by fitting the imaginary part of permittivity using a nonlinear least-squares approach. This analysis enables the electrical loss to be quantitatively separated into conduction loss and relaxation polarization loss under alternating electromagnetic fields. Owing to the continuous and mechanically stable network in the GA, its EM properties can be combined with subwavelength structures to introduce controllable magnetic responses and multiple resonances. In this way, electrical loss in the GA and structural resonances work together, leading to improved impedance matching and broadband absorption. As a result, the proposed GA-MMA exhibits a reflection loss (*RL*) below −10 dB over 9.1–49.8 GHz and maintains stable absorption under oblique incidence. This design offers a practical route to extend the bandwidth of GA-based absorbers by coupling material loss with structural effects, rather than relying on material optimization alone.

## 2. Materials and Methods

### 2.1. Reagents

The chemicals used in this study included graphene oxide (GO), which was supplied by Suzhou Tanfeng Graphene Technology Corporation, Suzhou, China. Other reagents, L-ascorbic acid (C_6_H_8_O_6_) and urea (CH_4_N_2_O), were procured from Shanghai Aladdin Corporation, Shanghai, China.

### 2.2. Synthesis of Graphene

Graphene was prepared through thermal reduction in a tube furnace with argon (Ar) protection. GO powder was evenly spread in a quartz boat and placed in the heating zone. High-purity argon (Ar) was introduced at a flow rate of 100 mL min^−1^ and maintained throughout the process. The temperature was increased at 10 °C min^−1^ to 800 °C and held for 10 s to rapidly deoxygenate GO, producing few-layer graphene with high purity while minimizing sheet restacking. The sample was then cooled down to room temperature under continuous Ar flow to obtain the target graphene.

### 2.3. Synthesis of Graphene Aerogel

The GA was synthesized by a low-temperature and ambient-pressure reduction process. Briefly, 50 mg of GO powder was dispersed in 25 mL of deionized water and subjected to ultrasonication for 30 min to form a homogeneous aqueous dispersion. Then, 50 mg of L-ascorbic acid and 1.25 g of urea were added to the GO dispersion as reducing agents, followed by an additional 30 min of ultrasonication to ensure uniform mixing. The resulting mixture was heated in an air oven at 90 °C for 10 h to obtain a reduced graphene hydrogel. Finally, the hydrogel was frozen at −70 °C and lyophilized for 36 h to yield the final GA samples.

### 2.4. Characterization

A comprehensive characterization was conducted to investigate the morphology, phase composition, and surface chemistry of the prepared graphene and graphene aerogel (GA). The microscopic morphology of graphene and the GA was observed using a scanning electron microscope (SEM, FEI Inspect F50, FEI Company, Hillsboro, OR, USA). Transmission electron microscopy (TEM) images were acquired using a JEM-F200 transmission electron microscope (JEOL Ltd., Tokyo, Japan) operated at an accelerating voltage of 200 kV to further reveal the internal microstructure. The crystalline phase structures were characterized by X-ray diffraction (XRD, Shimadzu Corporation, Kyoto, Japan). Raman spectra were recorded using a Raman spectrometer (XPLORA PLUS, Horiba Scientific, Kyoto, Japan) with a 532 nm Ar^+^ laser as the excitation source. The specific surface area and pore structure were determined by the Brunauer–Emmett–Teller (BET) method using an Autosorb iQ analyzer (Quantachrome Instruments, Boynton Beach, FL, USA). The surface chemical composition was examined by X-ray photoelectron spectroscopy (XPS, Kratos Analytical Ltd., Manchester, UK).

### 2.5. Electromagnetic Measurements

The graphene and GA samples were individually crushed and mixed with paraffin wax at mass ratios of 15 wt% and 10 wt%, respectively. And it was pressed into toroidal specimens (outer diameter: 7.00 mm; inner diameter: 3.00 mm). Their EM parameters were measured using a vector network analyzer (AV3629, CETC 41st Research Institute, Qingdao, China) over the frequency range of 2–18 GHz.

## 3. Results and Discussion

### 3.1. Analysis of the EMW Absorption Performance of Graphene Aerogel and Graphene

**Figure 1:**
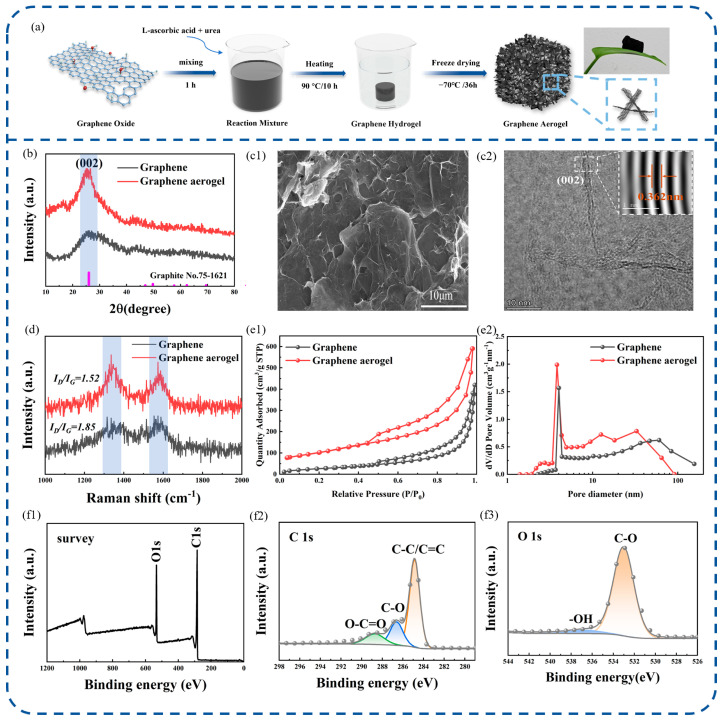
(**a**) Schematic illustration of the preparation process of the GA; (**b**) XRD patterns of the GA and graphene; (**c1**,**c2**) SEM and HRTEM images of the GA; (**d**) Raman spectrum; (**e1**) N_2_ adsorption–desorption isotherm; and (**e2**) BJH pore-size distribution. XPS analysis of the GA: (**f1**) survey spectrum; (**f2**) C 1s spectrum; and (**f3**) O 1s spectrum.

In this study, graphene and GA were prepared. As illustrated in Figure 1a, the GA was synthesized using L-ascorbic acid and urea as co-reducing agents under freeze-drying conditions. The obtained GA exhibits a self-supporting and ultralight structure with an extremely low bulk density, which was determined to be approximately 10.1 mg cm^−3^, allowing it to be easily picked up with tweezers without visible deformation. In addition, the GA shows good mechanical integrity and structural stability during handling and sample preparation, and all samples were prepared following the same synthesis protocol to ensure batch-to-batch consistency.

Following the preparation of the material samples, various analytical techniques were utilized to investigate their microstructural features. Figure 1b shows the XRD patterns of graphene and the GA, both exhibiting a characteristic (002) diffraction peak at 2θ ≈ 26.5°. This peak corresponds to the (002) plane of hexagonal graphite (PDF No. 75-1621), confirming that the samples were successfully synthesized with the typical layered graphene structure. Compared with graphene, the GA displays a narrower and more intense (002) peak, indicating higher graphitization and crystallinity. Figure 1(c1) shows the SEM image of the GA, which exhibits an intricate microstructure characterized by abundant pores, pronounced wrinkles, and distinct layer undulations. No apparent agglomeration is observed, contributing to the formation of a continuous 3D conductive network. Moreover, the highly wrinkled and porous architecture endows the GA with abundant interfacial regions, which are favorable for relaxation polarization loss. The microstructure of the graphene aerogel (GA) was further investigated by transmission electron microscopy. As shown in Figure 1(c2), the GA exhibits a highly wrinkled and disordered layered morphology, in which graphene sheets are loosely stacked and interconnected. High-resolution TEM reveals discernible lattice fringes with an interlayer spacing of approximately 0.362 nm, which can be indexed to the (002) plane of graphitic carbon. Notably, this interlayer spacing is larger than that of ideal graphite (0.335 nm), suggesting the presence of abundant structural defects and residual functional groups. Such defect-rich and expanded interlayer regions are expected to generate abundant interfacial polarization sites and relaxation polarization loss, which is consistent with the intricate porous morphology observed in SEM images. These microstructural features collectively confirm that the GA possesses a defect-rich, loosely stacked graphene network with enhanced interfacial regions, providing a favorable structural basis for improved electrical loss and broadband EMW attenuation. To gain deeper insights into the graphitization degree of the carbon framework, Raman spectroscopy was performed on both samples, as shown in Figure 1d. Both samples display two prominent peaks at approximately 1350 cm^−1^ and 1580 cm^−1^, which correspond to the D band and G band, respectively. The lower I_D_/I_G_ ratio of the GA indicates a reduced defect density and a higher degree of graphitization compared with graphene. Meanwhile, its 3D porous structure introduces abundant interfaces and residual functional groups, which promote interfacial and dipolar polarization, thereby enhancing relaxation loss.

Figure 1e shows the pore structure characteristics of the samples investigated by BET analysis. The GA exhibits a specific surface area of 364.17 m^2^ g^−1^, while graphene has a specific surface area of 205.23 m^2^ g^−1^ for graphene. In Figure 1(e1), the GA sample shows a typical type-IV nitrogen adsorption isotherm with a more pronounced hysteresis loop in the relatively high-pressure region compared with graphene, indicating a more developed porous structure and enhanced adsorption capacity. The Barrett–Joyner–Halenda (BJH) method pore-size distribution, as shown in Figure 1(e2), further reveals that the GA exhibits a distinct pore volume peak around 4–5 nm, whereas graphene shows a more dispersed pore volume distribution with a lower total pore volume. The SEM and BET analyses confirm that the GA possesses a more developed porous architecture, which in turn facilitates stronger relaxation polarization.

To further investigate the chemical composition and functional group distribution of the GA sample, XPS analysis was performed. Figure 1(f1) presents the XPS survey spectrum, confirming the presence of carbon (C) and oxygen (O) elements in the GA, and indicating a high abundance of oxygen-containing functional groups. In the C 1s spectrum shown in Figure 1(f2), the GA retains the same binding-energy components as graphene, dominated by C–C/C=C with minor contributions from C–O and O–C=O. In the O 1s spectrum shown in Figure 1(f3), the GA is primarily composed of C–O species, with a smaller contribution from –OH. High-resolution C 1s and O 1s spectra were deconvoluted, and the atomic percentages of individual chemical states were obtained directly from peak fitting. The results indicate that the GA consists of approximately 55.0 at.% carbon and 45.0 at.% oxygen. These features suggest that the GA exhibits a more heterogeneous distribution of oxygen-containing functional groups. In other words, due to the presence of oxygen-containing functional groups, the charge distribution in the GA becomes unbalanced, and as a result, there are many electric dipoles in the GA. The SEM and XPS characterization of graphene can be found in Figure S1 (Supporting Information) of the Supplementary Material.

To evaluate the microwave absorption performance, the *RL* of the GA was calculated based on the transmission line theory according to Equations (1) and (2) [34,35]:

(1) $$Z_{\mathrm{in}}=Z_{0}\sqrt{\frac{\mu_{r}}{\varepsilon_{r}}}\tanh\left(j\left(\frac{2\pi fd}{c}\sqrt{\mu_{r}\varepsilon_{r}}\right)\right),$$

(2) $$RL=20\log\left|\frac{\left(Z_{\mathrm{in}}-Z_{0}\right)}{\left(Z_{\mathrm{in}}+Z_{0}\right)}\right|.$$

where *Z_in_* and *Z*_0_ represent the input impedance of the absorbing layer and the characteristic impedance of free space, respectively. The parameters *c*, *f* and *d* correspond to the speed of light, the EMW frequency, and the sample thickness separately. Moreover, *ε_r_* and *μ_r_* denote the complex relative permittivity and permeability, respectively. The *RL* reflects the absorption capability of the material, where a lower *RL* value indicates stronger absorption. The EAB is defined as the frequency range in which *RL* remains below −10 dB.

**Figure 2:**
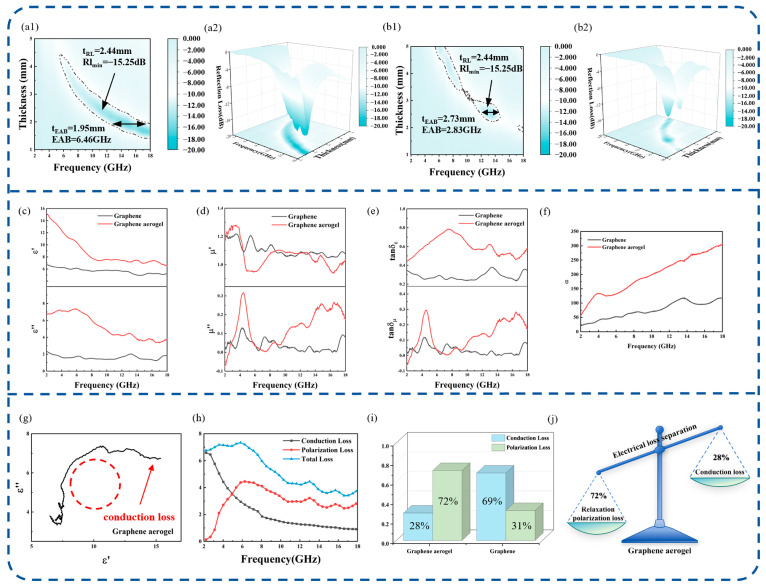
(**a1**,**a2**) The 2D and 3D RL–frequency curves of the GA. (**b1**,**b2**) The 2D and 3D RL–frequency curves of graphene. (**c**–**e**) The permittivity, permeability, and electrical/magnetic loss tangents. (**f**) Attenuation constant α of the GA and graphene. (**g**) Cole–Cole plots. (**h**) Quantitative separation of electrical losses in the GA. (**i**) Comparison of conduction loss and relaxation polarization losses within the EAB for graphene and the GA. (**j**) Schematic illustration of loss mechanism analysis.

Figure 2a,b present the RL contour of the GA in the 2–18 GHz range. The GA exhibits an EAB of 6.46 GHz at a matching thickness of 1.95 mm, covering 11.54–18.00 GHz, with a minimum *RLₘᵢₙ* of −15.3 dB at 10.77 GHz and 2.44 mm thickness. Comparatively, graphene shows a narrower EAB of 2.83 GHz at 2.73 mm and *RLₘᵢₙ* of −14.04 dB at 13.47 GHz (at 2.51 mm), confirming the superior absorption performance of the GA. Furthermore, with increasing thickness, the −10 dB contour gradually shifts to lower frequencies, which is consistent with the quarter-wavelength impedance-matching behavior. These results demonstrate that the GA achieves broad and stable absorption in the X–Ku band with a relatively small thickness.

A detailed analysis was conducted to gain deeper insight into the loss mechanisms of graphene and the GA by examining their measured EM parameters. To elucidate the EMW attenuation mechanism, the complex permittivity (*ε_r_* = *ε*′ − *jε*″) and permeability (*μ_r_* = *μ*′ − *jμ*″) were examined. The real components (ε′, *μ*′) represent the capacity to store EM energy, whereas the imaginary components (*ε*″, *μ*″) correspond to energy dissipation within the absorbing material. The electrical and magnetic loss tangents (tan *δ_ε_* = *ε*″/*ε*′, tan *δ_μ_* = *μ*″/*μ*′) were further employed to quantify the respective loss contributions, with tan *δ_μ_* reflecting the magnetic dissipation capability. Before discussing the frequency-dependent EM response in detail, it is noted that for EM measurements, each sample was subjected to repeated measurements under identical conditions. The materials and absorber designs investigated in this work are intended for typical radar operating environments, which are generally close to normal temperature. Accordingly, the EM parameters were measured at normal temperature, and the corresponding EM characterization and simulations were performed under these representative conditions. As displayed in Figure 2c, both samples show a clear frequency-dependent variation in permittivity. The permittivity values remain relatively high at lower frequencies and then gradually decrease with increasing frequency, mainly due to the relaxation of dipolar and interfacial polarizations. Throughout the 2–18 GHz range, the GA exhibits considerably larger *ε*′ and *ε*″ than graphene, revealing its enhanced ability to store and dissipate EM energy. This improvement arises from the 3D porous structure of the GA, where interconnected conductive pathways and numerous interfaces promote interfacial and dipolar polarization. As shown in Figure 2d, both materials exhibit a real part of permeability (*μ*′) close to unity and an imaginary part (*μ*″) approaching zero. It demonstrates that the EM loss is primarily governed by electrical loss mechanisms under high-frequency EM fields. Figure 2e presents a comparative analysis between the electrical loss tangent (tanδ_ε_) and magnetic loss tangent (tan*δ_μ_*) of graphene and the GA across 2–18 GHz. The GA sample shows a much higher tan*δ_ε_* than graphene, and the value gradually declines with frequency, reflecting typical relaxation of dipolar and interfacial polarization. This enhancement mainly arises from the 3D conductive framework and numerous internal interfaces in the GA, which strengthen conduction loss and polarization relaxation. In contrast, tan*δ_μ_* remains relatively low for both materials, with the GA exhibiting only small oscillation, implying that magnetic loss contributes little to the overall attenuation. Throughout the measured band, tan*δ_ε_* consistently exceeds tan*δ_μ_*, confirming that electrical dissipation is the primary mechanism governing EM-energy loss in the GA.

The attenuation constant (*α*) is a crucial factor that influences the absorption performance of a material. It represents the intrinsic energy dissipation capability of the absorbing layer, and its corresponding expressions are given below [36,37]:

(3) $$\alpha =\frac{\sqrt{2\pi f}}{c}\left[\sqrt{\left(\mu^{\prime \prime }\varepsilon^{\prime \prime }-\mu^{\prime }\varepsilon^{\prime }\right)+\sqrt{{\left(\mu^{\prime }\varepsilon^{\prime \prime }+\mu^{\prime \prime }\varepsilon^{\prime }\right)}^{2}+{\left(\mu^{\prime \prime }\varepsilon^{\prime \prime }-\mu^{\prime }\varepsilon^{\prime }\right)}^{2}}}\right]$$

As shown in Figure 2e, a higher α value corresponds to stronger EMW attenuation. For both samples, *α* increases with frequency, with the GA consistently maintaining much higher α values throughout the 2–18 GHz range, indicating its superior ability to dissipate EM energy. The higher *ε*″, together with a significantly larger tan*δ_ε_* compared to tan*δ_μ_*, suggests that electrical loss mechanisms play a dominant role in the overall absorption behavior.

In the GA, electrical loss primarily originates from polarization relaxation and conductive dissipation. To further analyze the contribution of these mechanisms, the Debye relaxation model was adopted. According to Debye’s theory, the relationship between the real and imaginary parts of complex permittivity can be expressed as follows [38,39]:

(4) $${\left(\varepsilon^{\prime }-\frac{\varepsilon_{s}+\varepsilon_{\infty }}{2}\right)}^{2}+{\left(\varepsilon^{\prime \prime }\right)}^{2}={\left(\frac{\varepsilon_{s}-\varepsilon_{\infty }}{2}\right)}^{2}$$

Based on the Debye relaxation analysis, the electrical response of the GA can be further interpreted through its Cole–Cole plot, as shown in Figure 2g. The plot displays a broad semicircular arc followed by a short linear tail at higher *ε*″ values. This semicircular region reflects relaxation processes with a distribution of relaxation times, primarily stemming from dipolar polarization of residual oxygen-containing groups and interfacial polarization within the 3D porous framework. In contrast, the appearance of the linear tail indicates the contribution of conduction loss, which originates from the interconnected conductive network formed by overlapping graphene sheets. These combined features demonstrate that the electrical loss of the GA results from the synergistic effect of polarization relaxation and electrical conduction, consistent with its relatively high *ε*″ and tan *δₑ* values over the 2–18 GHz range. By fitting the data to the Debye model using Equations (5) and (6), the relationship can be linearized as shown in Equation (7) [39,40]:

(5) $$\varepsilon^{\prime }=\varepsilon_{\infty }+\frac{\varepsilon_{s}-\varepsilon_{\infty }}{1+{\left(2\pi f\tau \right)}^{2}},$$

(6) $$\varepsilon^{\prime \prime }=\frac{\left(\varepsilon_{s}-\varepsilon_{\infty }\right)\omega \tau }{1+{\left(\omega \tau \right)}^{2}}+\frac{\sigma }{\omega \varepsilon_{0}},$$

(7) $$\varepsilon^{\prime }\approx \frac{1}{2\pi f}\varepsilon^{\prime \prime }+\varepsilon_{\infty }.$$

The slope of the linear fitting indicates the relaxation rate, which is proportional to 1/*τ*. Figure 2h illustrates the quantitively classified electrical losses by our group [41]. In previous studies, quantitative analysis of electrical loss is often simplified by treating the electrical conductivity as a frequency-independent parameter. However, according to classical dielectric relaxation theory, the effective conductivity under alternating electromagnetic fields is inherently frequency dependent. Such simplifications may obscure the relative contributions of conduction loss and polarization-related loss processes. To quantitatively deconvolute each loss contributions, the imaginary permittivity *ε*″ was fitted using a nonlinear least-squares method and separated into relaxation polarization loss (*ε_p_*″) and conductive loss (*ε_c_*″), providing clear guidance for mechanism analysis and structural optimization. In the low-frequency range of 2–5 GHz, conduction loss dominates, while at higher frequencies, relaxation polarization becomes the main energy dissipation mechanism. As shown in Figure S2a (Supporting Information), analysis of the relaxation timescales reveals three distinct polarization processes in the GA. The low-frequency region corresponds to a slow relaxation process (*τ* > 0.3 ns). The intermediate-frequency region shows a moderate relaxation process (*τ* ≈ 0.5–1 ns). The high-frequency region is associated with a fast relaxation process (*τ* < 0.2 ns). For clarity, these three time-scale ranges are denoted as *τ*_1_ (slow), *τ*_2_ (medium-timescale), and *τ*_3_ (fast). The slow relaxation (*τ*_1_) arises from interfacial polarization occurring at sheet junctions and pore boundaries within the 3-D network. The medium-timescale relaxation process (*τ*_2_) is associated with charge accumulation and polarization within the porous conductive framework. The fast relaxation (*τ*_3_) corresponds to the reorientation of dipoles related to oxygen-containing groups and structural defects. These multiple relaxation processes collectively contribute to the enhanced electrical response and energy dissipation capability of the GA. The relationship between ε′ versus *ε*″/*f* of graphene and the GA in Figure S2a,b (Supporting Information). The Cole–Cole plots and the quantitative separation of electrical losses for graphene are shown in Figure S3a,b (Supporting Information). The impedance matching and quarter-wavelength thickness curves of graphene and the GA are shown in Figure S4a–d (Supporting Information).

In addition to the EM parameters, impedance matching governs the coupling efficiency between the incident wave and the absorbing material. The attenuation constant α is calculated according to Equation (3), and the impedance mismatch factor |Δ| is expressed as [42,43]:

(8) $$|\Delta |=\left|\sinh^{2}\left(Kfd\right)-M\right|.$$

*K* and *M* were calculated by using Equations (9) and (10), as follows:

(9) $$K=\frac{4\pi \sqrt{\mu^{\prime \prime }\varepsilon^{\prime \prime }}\sin\left(\frac{\delta_{e}+\delta_{\mu }}{2}\right)}{c\cos\delta_{e}\cos\delta_{\mu }},$$

(10) $$M=\frac{4{\varepsilon^{\prime }}_{r}\cos\delta_{e}{\mu^{\prime }}_{r}\cos\delta_{\mu }}{{\left(\mu^{\prime }\cos\delta_{e}-\varepsilon^{\prime }\cos\delta_{\mu }\right)}^{2}+{\left[\tan\left(\frac{\delta_{\mu }}{2}\frac{\delta_{e}}{2}\right)\right]}^{2}{\left(\mu^{\prime }\cos\delta_{e}+\varepsilon^{\prime }\cos\delta_{\mu }\right)}^{2}}.$$

A smaller |Δ| value indicates better impedance matching with free space (377 Ω). When |Δ| stays below 0.4 across a wide frequency range, the absorbing material can be deemed to possess favorable impedance-matching characteristics. As illustrated in Figure S4a,b. The effective matching region (|Δ| < 0.4) of the GA accounts for 35.01% of the total range, which is more than three times that of graphene (11.37%). The matching zone of the GA extends smoothly from lower frequencies at greater thicknesses to higher frequencies at thinner layers, demonstrating broad and stable impedance matching. In contrast, graphene shows only small and discontinuous matching areas, mainly located in the high-frequency region and necessitating thicker samples. Overall, the absorption behavior is governed by the balance between attenuation and impedance matching. The GA achieves a favorable compromise, combining strong energy-dissipation ability with broad impedance matching. Consequently, it allows efficient wave entry and loss even at reduced thickness.

Analysis of the *RL* data shows a clear inverse trend between the optimal absorption thickness and the corresponding frequency. With the increase in the matching thickness, the frequency of the minimum *RL* gradually shifts to lower values. This phenomenon follows the quarter-wavelength (λ/4) interference model, which can be expressed as follows [44]:

(11) $$d_{m}=\frac{n\lambda }{4}=\frac{nc}{4f_{m}\sqrt{\left|\varepsilon_{r}||\mu_{r}\right|}}\;\left(n=1,3,5,\ldots \right)$$

As shown in Figure S4c,d, the RL curves of the GA at different thicknesses exhibit a clear frequency-dependent shift. With increasing thickness, the *RLₘᵢₙ* points move gradually toward lower frequencies, consistent with the quarter-wavelength (*λ*/4) cancellation model. According to Equation (12), the matching thickness dm and the corresponding frequency *f_m_* satisfy the *λ*/4 phase condition, where destructive interference minimizes reflection. Experimentally, the *RLₘᵢₙ* reaches −15.3 dB at 10.77 GHz with a thickness of 2.44 mm, aligning closely with the theoretical *λ*/4 prediction. This agreement demonstrates that phase interference is the primary mechanism of absorption. Therefore, the GA structure achieves good impedance matching and effectively dissipates the incident EMWs within the X–Ku band.

### 3.2. Design and Optimization of the GA-MMA

**Figure 3:**
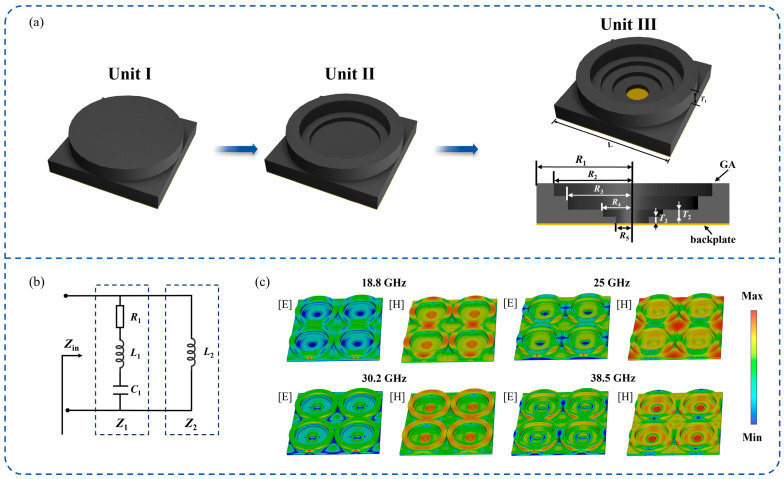
(**a**) Design process; (**b**) equivalent circuit; and (**c**) electromagnetic field distributions at representative frequencies.

Next, an MMA was designed based on the prepared GA, referred to as GA-MMA. The experimentally measured electromagnetic parameters of graphene and graphene aerogel in the 2–18 GHz range were imported into CST Microwave Studio to define a dispersive material model. It should be noted that the electromagnetic parameters above 18 GHz were not directly measured, but obtained through causal dispersion fitting and model-based extrapolation based on the measured data. As shown in Figure 3a, the unit-cell of the proposed GA-MMA comprises a top GA layer and a bottom metallic backplate. The key distinction between the GA-MMA and the traditional MAM-type MA is that the metal pattern in the traditional MA is replaced by a medium of a certain thickness. On the one hand, the unit-cell of MMA functions as a resonator, converting the incident EMW into a standing wave. On the other hand, the medium pattern carries the function of the traditional absorbing coating and consumes the EMW through rich physical mechanisms. This is the fundamental reason why GA-MMA achieves ultra-wideband high-efficiency absorption under the GA-MMA.

The unit-cell design evolved through three stages. Initially, Unit I consisted of cylindrical structures stacked on a square substrate. In the second stage, the cylinder was replaced by two concentric rings with different inner radii, forming Unit II. To further enhance bandwidth, two circular apertures were added at the center of the substrate to obtain Unit III. This configuration represents the final design of the unit-cell, with detailed structural parameters listed in Table 1. It should be noted that the proposed GA-MMA requires a metallic backplate during operation, typically provided by the installation platform and not a part of the design.

MAs are essentially circuit-assisted EMW absorption devices. The equivalent circuit of the proposed unit-cell is shown in Figure 3b. When an EMW is normally incident on the MMA surface, the induced surface current on the GA corresponds to an inductance *L*_1_, while the gap between adjacent units is represented by a capacitance *C*_1_. The electrical loss is expressed as a resistance *R*_1_, and the surface current on the metallic backplate is modeled as an inductance *L*_2_. Accordingly, the input impedance *Z_in_* of the GA-MMA can be expressed as [45,46]:

(12) $$\left\{\begin{array}{l} Z_{1}=R_{1}+j\left(\omega L_{1}-\frac{1}{\omega C_{1}}\right)\\ Z_{2}=j\omega L_{2}\\ Z_{\mathrm{in}}=Z_{1}//Z_{2}=\frac{R_{1}\omega^{2}L_{2}^{2}+j\omega L_{2}\left(R_{1}^{2}+X_{1}^{2}+X_{1}\omega L_{2}\right)}{R_{1}^{2}+{\left(X_{1}+\omega L_{2}\right)}^{2}} \end{array}\right.$$

Clearly, the optimization of the metamaterial unit structure fundamentally involves the regulation of equivalent circuit parameters, such as *R*, *L* and *C*. Consequently, the input impedance of the GA-MMA is designed to satisfy the following condition [47]:

(13) ReZin=η0ImZin=0

where *η*_0_ denotes the intrinsic impedance of free space. Under this condition, impedance matching is achieved between the GA-MMA and free space. Simultaneously, the EMW within a specific frequency band resonates within the absorber, significantly facilitating the dissipation of EM energy.

**Figure 4:**
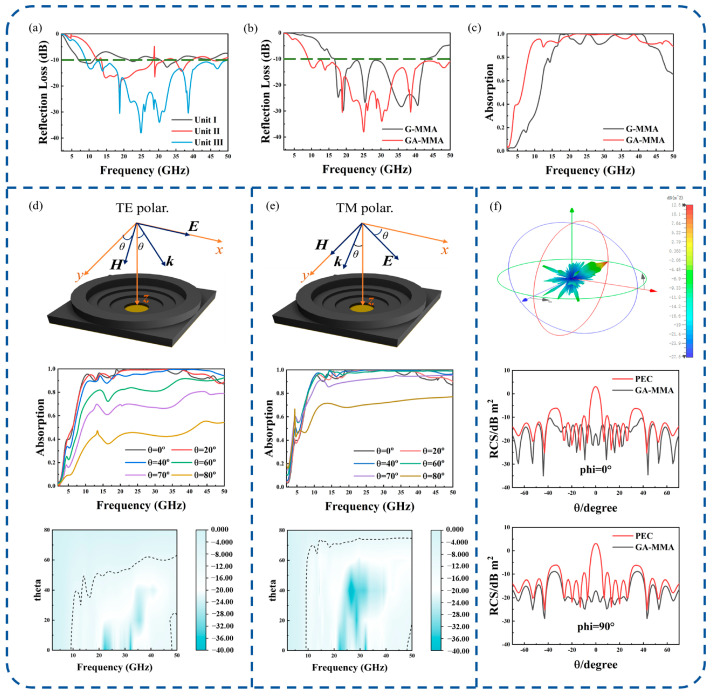
(**a**) The RL curves of three unit-cells. The green dashed line indicates the −10 dB reflection loss threshold for effective absorption. Comparative analysis between the GA-MMA and G-MMA: (**b**) RL; (**c**) absorptance. Absorption performance of the GA-MMA under oblique incidence: (**d**) TE polarization; (**e**) TM polarization. (**f**) Simulated RCS characteristics of the GA-MMA.

To further verify the absorption mechanism, the internal electric and magnetic field distributions of the GA-MMA were simulated, as shown in Figure 3c. The measured EM parameters of graphene and the GA were imported into CST Microwave Studio for accurate modeling. The four analyzed frequencies (18.8, 25, 30.2, and 38.5 GHz) correspond to the strongest absorption peaks in the reflection-loss curve of Figure 4b. The E-field is mainly concentrated at the electrical interfaces, indicating strong capacitive coupling and charge accumulation within the GA framework. In contrast, the H-field exhibits distinct circular loops confined within the ring structures, confirming the presence of localized magnetic resonance modes. This clearly demonstrates that the ring geometry effectively introduces magnetic responses into the nonmagnetic GA-based system. The coexistence of multiple magnetic resonances at these frequencies, combined with the intrinsic electrical loss of the GA, produces synergistic energy dissipation across a broad frequency range, which accounts for the observed ultra-broadband absorption performance.

Figure 4a presents the RL curves of the three unit-cells. Evidently, as the structure undergoes progressive optimization, the *RL* of the GA-MMA decreases gradually, and the impedance bandwidth expands correspondingly. Furthermore, when the medium material is changed from the GA to graphene while keeping the structural configuration the same, the *RL* and absorptance (*A*) of the two configurations are compared. The *A* can be represented as [48,49]:

(14) $$A=1-|S_{11}{|}^{2}-|S_{21}{|}^{2}$$

where *S*_11_ and *S*_21_ denote the reflection coefficient and transmission coefficient of the absorber, respectively. In this design, the presence of the metallic backplate ensures that the *S*_21_ is negligible, thereby enhancing overall absorption performance.

Figure 4b,c illustrate that the bandwidth of the graphene-based metamaterial absorber (G-MMA), with *RL* < −10 dB and absorption > 90%, spans 16.54–43.04 GHz. Comparatively, the bandwidth of the GA-MMA ranges from 9.1 to 49.8 GHz. The absorption peaks reach 99.9% at 18.8, 25, 30.2, and 38.5 GHz. Replacing graphene with the GA markedly enhances the absorption performance of MMA in the low-frequency range and further broadens the bandwidth.

Figure 4d,e illustrate the influence of incident angle (θ) on the absorption performance of the GA-MMA under transverse electric (TE) and transverse magnetic (TM) modes. They show the EM field configurations, absorptance variation with θ, and 2D contour maps of θ, frequency, and absorptance. In TE polarization, the incident electric field *E* is aligned with the *x*-axis (perpendicular to the plane of incidence), while the magnetic field *H* and wave vector *k* rotate with θ. In TM polarization, *H* is aligned with the *y*-axis, and *E* and k vary with θ. With increasing θ, the absorptance of the GA-MMA decreases, yet even at θ = 70°, the structure maintains > 50% absorptance over 9.1–49.8 GHz, demonstrating robust wide-angle stability.

Finally, a 5 × 5 array was constructed by using the designed GA-MMA unit, and the radar cross-section (RCS) was simulated. The array size was 120 mm × 120 mm. As shown in Figure 4f, demonstrates that at 25 GHz, the RCS values of the GA-MMA are significantly lower than those of an ideal perfect electric conductor (PEC) within ±30° incidence. Moreover, within this angular range, the RCS curve of the GA-MMA exhibits excellent smoothness, indicating improved adaptability to variations in incidence angle.

**Figure 5:**
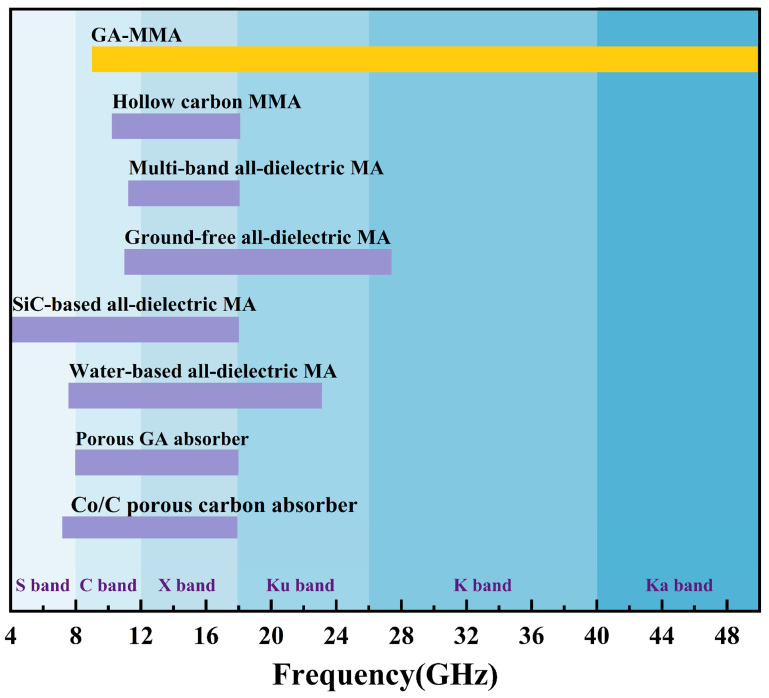
Comparison of the −10 dB EAB between the proposed GA-MMA and representative absorbers reported in the literature, plotted across standard microwave bands. References: [19,20,21,22,23,24,25].

Figure 5 compares the −10 dB absorption bandwidth of the proposed GA-MMA with representative absorbers reported in the literature. Most previously reported structures exhibit effective absorption confined to relatively narrow frequency ranges. By contrast, the GA-MMA shows continuous broadband absorption spanning several standard microwave bands. This comparison suggests that coupling the intrinsic electrical loss of graphene aerogel with subwavelength structural design is effective for extending the absorption bandwidth.

**Figure 6:**
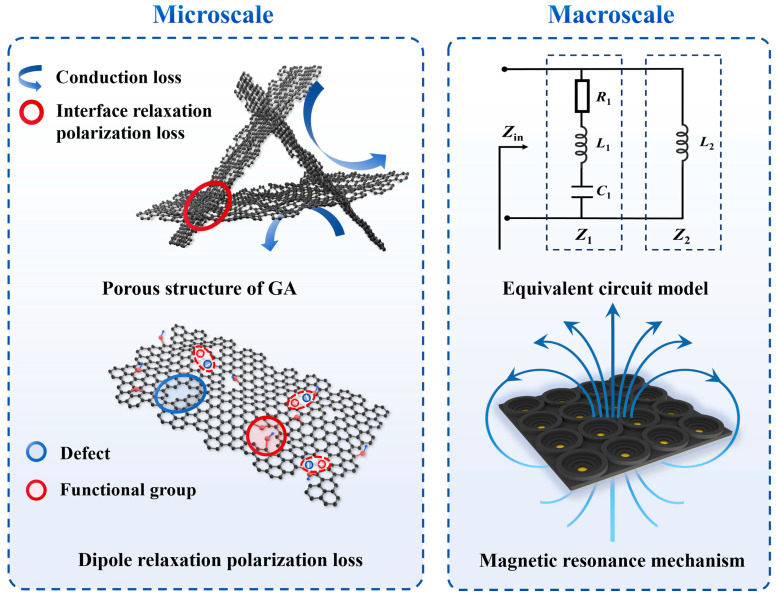
Schematic of the multiscale absorption mechanisms in the prepared GA-MMA.

The EMW absorption mechanism of the GA-MMA is illustrated in Figure 6. At the microstructural level, the 3D porous GA provides a continuous conductive network that facilitates charge transport and enhances conduction loss. The interconnected graphene sheets and pore interfaces create abundant homogeneous and heterogeneous junctions, where charge accumulation gives rise to interfacial relaxation polarization. In addition, the residual oxygen-containing functional groups and structural defects in the graphene framework act as dipoles, inducing dipole relaxation polarization. These polarization processes, together with electronic conduction, jointly dissipate the incident EM energy and convert it into heat through multiscale electrical loss.

At the macroscopic scale, the GA-MMA can be regarded as an equivalent RLC circuit absorber. The induced surface current on the GA layer corresponds to inductance (*L*_1_), the spacing between adjacent units behaves as capacitance (*C*_1_), the electrical loss is represented by resistance (*R*_1_), and the metallic backplane contributes an additional inductance (*L*_2_). Through careful adjustment of these equivalent parameters, the input impedance (*Z_in_*) can match the free-space impedance (*η*_0_), achieving efficient impedance matching and minimizing reflection. Moreover, the ring-shaped resonant unit introduces magnetic resonance, compensating for the intrinsic nonmagnetic nature of the GA and further enhancing the loss mechanisms.

This coupling between microscopic electrical losses and macroscopic EM resonances enables efficient attenuation of incident waves over a broad frequency range, allowing the GA-MMA to achieve broadband, stable, and lightweight absorption performance.

## 4. Conclusions

In summary, the broadband, lightweight, and wide-angle absorption performance of the proposed GA-MMA has been numerically demonstrated. To elucidate its EMW attenuation mechanism, a dual-scale analytical framework was constructed by coupling the microscopic loss behavior of the graphene aerogel with the macroscopic structure-induced magnetic response in the metamaterial unit-cell. At the microscopic level, graphene aerogel primarily exhibits electrical losses arising from conduction loss and relaxation polarization. To achieve quantitative insight into these loss processes, the imaginary part of permittivity was analyzed using a nonlinear least-squares fitting approach. In the GA, the average contribution of conductive loss within the effective absorption bandwidth is quantified as 28.2%, whereas relaxation polarization loss accounts for 71.8%, providing quantitative guidance for the mechanism-oriented design of EMW-absorbing materials. To compensate for the intrinsically weak magnetic response of the graphene aerogel, a ring-type resonant unit was introduced and further refined through iterative structural optimization to enhance magnetic coupling. Benefiting from the cooperative effect between material loss and structure-induced resonance, the GA-MMA exhibits a simulated minimum reflection loss of −38 dB at 25 GHz and achieves a wide effective absorption bandwidth of 40.7 GHz (9.1–49.8 GHz, *RL* < −10 dB), which is markedly broader than that of pristine graphene aerogel. The equivalent-circuit analysis suggests LC-type resonance behavior, and simulated magnetic field distributions support the presence of localized magnetic responses, consistent with improved impedance matching and enhanced energy dissipation. Overall, this study presents a material–structure coupling strategy for MMAs, providing a feasible design route for extending the absorption bandwidth of graphene-based absorbents beyond material-only limitations. The results highlight the potential of combining intrinsic electrical loss with tailored subwavelength resonant structures for the development of lightweight and broadband EMW absorbing materials. It should be noted that the absorption performance of the GA-MMA is currently demonstrated through numerical simulations, and experimental validation of the metamaterial absorber itself remains a subject of future work. Nevertheless, the present study provides a solid theoretical and design foundation for subsequent experimental realization and performance verification.

## Figures and Tables

**Table 1:** Structural parameters of the proposed GA-MMA (in mm).

L	T_1_	T_2_	T_3_	R_1_	R_2_	R_3_	R_4_	R_5_
24	2.5	1.5	0.035	12	9	8	3	2

## Data Availability

The data that support the findings of this study are available from the corresponding authors upon reasonable request.

## Supplementary Materials

The following supporting information can be downloaded at https://www.mdpi.com/article/10.3390/nano16010018/s1. Figure S1: SEM images and XPS spectra of graphene. Figure S2: Relationships between ε′ and ε″/f for graphene and graphene aerogel. Figure S3: Cole–Cole plots and quantitative separation of electrical losses for graphene. Figure S4: Impedance matching characteristics and quarter-wavelength thickness curves of graphene and graphene aerogel. Table S1: Quantitative classification of electrical losses for graphene and graphene aerogel.

## Abbreviations

The following abbreviations are used in this manuscript:

MMA	All-medium metamaterial absorber
GA	Graphene aerogel
EMW	Electromagnetic wave
EAB	Effective absorption bandwidth
GA-MMA	GA-based MMA
RL	Reflection loss

## References

[1] Cheng J., Li C., Xiong Y., Zhang H., Raza H., Ullah S., Wu J., Zheng G., Cao Q., Zhang D. (2022). Recent advances in design strategies and multifunctionality of flexible electromagnetic interference shielding materials. Nano-Micro Lett..

[2] Kim S.H., Lee S.Y., Zhang Y., Park S.J., Gu J. (2023). Carbon-based radar absorbing materials toward stealth technologies. Adv. Sci..

[3] Chen G., Li Z., Zhang L., Chang Q., Chen X., Fan X., Chen Q., Wu H. (2024). Mechanisms, design, and fabrication strategies for emerging electromagnetic wave-absorbing materials. Cell Rep. Phys. Sci..

[4] Korkmaz E., Aerts S., Coesoij R., Bhatt C.R., Velghe M., Colussi L., Land D., Petroulakis N., Spirito M., Bolte J. (2024). A comprehensive review of 5G NR RF-EMF exposure assessment technologies: Fundamentals, advancements, challenges, niches, and implications. Environ. Res..

[5] Xiong C., Xiong Q., Zhao M., Wang B., Dai L., Ni Y. (2023). Recent advances in non-biomass and biomass-based electromagnetic shielding materials. Adv. Compos. Hybrid Mater..

[6] Isari A.A., Ghaffarkhah A., Hashemi S.A., Wuttke S., Arjmand M. (2024). Structural design for EMI shielding: From underlying mechanisms to common pitfalls. Adv. Mater..

[7] Xiao J., He M., Zhan B., Guo H., Yang J.-l., Zhang Y., Qi X., Gu J. (2024). Multifunctional microwave absorption materials: Construction strategies and functional applications. Mater. Horiz..

[8] Lan D., Hu Y., Wang M., Wang Y., Gao Z., Jia Z. (2024). Perspective of electromagnetic wave absorbing materials with continuously tunable effective absorption frequency bands. Compos. Commun..

[9] Li W., Xu M., Xu H.X., Wang X., Huang W. (2022). Metamaterial absorbers: From tunable surface to structural transformation. Adv. Mater..

[10] Wang B.X., Xu C., Duan G., Xu W., Pi F. (2023). Review of broadband metamaterial absorbers: From principles, design strategies, and tunable properties to functional applications. Adv. Funct. Mater..

[11] Kim M., Jung K., Choi Y., Hwang S.S., Hyun J.K. (2022). Coupled solid and inverse antenna stacks above metal ground as metamaterial perfect electromagnetic wave absorbers with extreme subwavelength thicknesses. Adv. Opt. Mater..

[12] Yu H., Liang Z., Shi X., Yang F., Xu H., Wu Z., Dai R., Smith D.R., Liu Y. (2024). Long-Infrared Broadband Polarization-Sensitive Absorber with Metasurface Based on Ladder Network. Adv. Opt. Mater..

[13] Wang K., Li J., Han X., Qu L., Jin X., Ran Y., Geng Y., Chen H., Yi Y., Zhang Q. (2025). Ultrawideband microwave metamaterial absorber with excellent absorption performance and high optical transparency based on double-layer mesh structures. Opt. Express.

[14] Liu Y., Li C., Tang Y., Meng X., Zou X., Fang B., Hong Z., Jing X. (2024). Terahertz wave all-dielectric broadband tunable metamaterial absorber. J. Light. Technol..

[15] Landy N.I., Sajuyigbe S., Mock J.J., Smith D.R., Padilla W.J. (2008). Perfect metamaterial absorber. Phys. Rev. Lett..

[16] Watts C.M., Liu X., Padilla W.J. (2012). Metamaterial electromagnetic wave absorbers. Adv. Mater..

[17] Aydin K., Ferry V.E., Briggs R.M., Atwater H.A. (2011). Broadband polarization-independent resonant light absorption using ultrathin plasmonic super absorbers. Nat. Commun..

[18] Wang B.X., Qin X., Duan G., Yang G., Huang W.Q., Huang Z. (2024). Dielectric-based metamaterials for near-perfect light absorption. Adv. Funct. Mater..

[19] Cao M., Huang X., Gao L., Li X., Guo L., Yang H. (2022). Broadband bi-directional all-dielectric transparent metamaterial absorber. Nanomaterials.

[20] Hao D., Liu J., Zou P., Zhang Y., Moro R., Ma L. (2024). All-dielectric Metasurfaces and Their Applications in the Terahertz Range. Laser Photonics Rev..

[21] Sui J., Zhang J., Yan K., Guo J., Li S., Liu J., Yun J., Kang P., Ren Y., Zhang H. (2025). Breaking bandwidth limit: All-medium metamaterial absorber engineered from heterostructure-anchored N-doped hollow carbon spheres. Chem. Eng. J..

[22] Zhang F., Wang Q., Zhou T., Xiong Y., Wen Y., Jiang C., Wang Y., Du Z., Abrahams I., Wang L. (2020). A multi-band binary radar absorbing metamaterial based on a 3D low-permittivity all-dielectric structure. J. Alloys Compd..

[23] Xie J., Quader S., Xiao F., He C., Liang X., Geng J., Jin R., Zhu W., Rukhlenko I.D. (2019). Truly all-dielectric ultrabroadband metamaterial absorber: Water-based and ground-free. IEEE Antennas Wirel. Propag. Lett..

[24] Li W., Li C., Lin L., Wang Y., Zhang J. (2019). All-dielectric radar absorbing array metamaterial based on silicon carbide/carbon foam material. J. Alloys Compd..

[25] Chen Z., Shen Z., Liu H., Shu X. (2024). Ultra-broadband transmission absorption of the all-dielectric water-based metamaterial. Int. J. Appl. Electromagn. Mech..

[26] Meng Y., Chen Y., Lu L., Ding Y., Cusano A., Fan J.A., Hu Q., Wang K., Xie Z., Liu Z. (2021). Optical meta-waveguides for integrated photonics and beyond. Light Sci. Appl..

[27] Lin K.-T., Lin H., Yang T., Jia B. (2020). Structured graphene metamaterial selective absorbers for high efficiency and omnidirectional solar thermal energy conversion. Nat. Commun..

[28] Shu R., Wang C., Xu L., Guan Y., Tian K. (2025). Synthesis of layered double hydroxide derivative-decorated nitrogen-doped graphene composite aerogels with a unique hierarchical porous network structure for microwave absorption. J. Mater. Chem. C.

[29] Zhong Y., Long Y., Sun Y., Qin J., Li Y., Liang G., Zou J., Xie P. (2025). Ultra-lightweight carbon nanocomposites as microwave absorber with high absorbing performance derived from flour. Adv. Compos. Hybrid Mater..

[30] Nie Z., Feng Y., Hu X., Su J., Zhao Z., Chen J., Wang R., Qi S. (2023). In situ-growth ultrathin hexagonal boron nitride/N-doped reduced graphene oxide composite aerogel for high performance of thermal insulation and electromagnetic wave absorption. Ceram. Int..

[31] Shu R., Xu J., Shi J. (2023). Synthesis of nitrogen-doped graphene-based binary composite aerogels for ultralightweight and broadband electromagnetic absorption. Ceram. Int..

[32] Hou Y., Sheng Z., Fu C., Kong J., Zhang X. (2022). Hygroscopic holey graphene aerogel fibers enable highly efficient moisture capture, heat allocation and microwave absorption. Nat. Commun..

[33] Tian H., Lin J., Liu J., Li L., Li B., Zheng S., Liu W., Liu C., Zeng Z., Wu N. (2024). Ultralight SiO_2_ nanofiber-reinforced graphene aerogels for multifunctional electromagnetic wave absorber. ACS Appl. Mater. Interfaces.

[34] Yang W., Jiang B., Che S., Yan L., Li Z.-x., Li Y.-f. (2021). Research progress on carbon-based materials for electromagnetic wave absorption and the related mechanisms. New Carbon Mater..

[35] Cui L., Han X., Wang F., Zhao H., Du Y. (2021). A review on recent advances in carbon-based dielectric system for microwave absorption. J. Mater. Sci..

[36] Ma H., Fashandi M., Rejeb Z.B., Ming X., Liu Y., Gong P., Li G., Park C.B. (2024). Efficient electromagnetic wave absorption and thermal infrared stealth in PVTMS@ MWCNT nano-aerogel via abundant nano-sized cavities and attenuation interfaces. Nano-Micro Letters.

[37] Li F., Wu N., Kimura H., Wang Y., Xu B.B., Wang D., Li Y., Algadi H., Guo Z., Du W. (2023). Initiating binary metal oxides microcubes electromagnetic wave absorber toward ultrabroad absorption bandwidth through interfacial and defects modulation. Nano-Micro Lett..

[38] Ma M., Bi Y., Tong Z., Liu Y., Lyu P., Wang R., Ma Y., Wu G., Liao Z., Chen Y. (2021). Recent progress of MOF-derived porous carbon materials for microwave absorption. RSC Adv..

[39] Wang C., Murugadoss V., Kong J., He Z., Mai X., Shao Q., Chen Y., Guo L., Liu C., Angaiah S. (2018). Overview of carbon nanostructures and nanocomposites for electromagnetic wave shielding. Carbon.

[40] Lunkenheimer P., Krohns S., Riegg S., Ebbinghaus S.G., Reller A., Loidl A. (2009). Colossal dielectric constants in transition-metal oxides. Eur. Phys. J. Spec. Top..

[41] Wang J., Zhang L., Yan J., Yun J., Zhao W., Dai K., Wang H., Sun Y. (2024). MXene-based ultrathin electromagnetic wave absorber with hydrophobicity, anticorrosion, and quantitively classified electrical losses by intercalation growth nucleation engineering. Adv. Funct. Mater..

[42] Zhang D., Liu T., Cheng J., Cao Q., Zheng G., Liang S., Wang H., Cao M.-S. (2019). Lightweight and high-performance microwave absorber based on 2D WS2–RGO heterostructures. Nano-Micro Lett..

[43] Zhang Z., Cai Z., Wang Z., Peng Y., Xia L. (2021). A review on metal-organic framework-derived porous carbon-based novel microwave absorption materials. Nano-Micro Lett..

[44] Zhang S., Wang T., Gao M., Wang P., Pang H., Qiao L., Li F. (2020). Strict proof and applicable range of the quarter-wavelength model for microwave absorbers. J. Phys. D Appl. Phys..

[45] Hannan S., Islam M.T., Faruque M.R.I., Chowdhury M.E., Musharavati F. (2021). Angle-insensitive co-polarized metamaterial absorber based on equivalent circuit analysis for dual band WiFi applications. Sci. Rep..

[46] Hokmabadi M.P., Wilbert D.S., Kung P., Kim S.M. (2013). Design and analysis of perfect terahertz metamaterial absorber by a novel dynamic circuit model. Opt. Express.

[47] Shen X., Yang Y., Zang Y., Gu J., Han J., Zhang W., Jun Cui T. (2012). Triple-band terahertz metamaterial absorber: Design, experiment, and physical interpretation. Appl. Phys. Lett..

[48] Lee N., Kim T., Lim J.-S., Chang I., Cho H.H. (2019). Metamaterial-selective emitter for maximizing infrared camouflage performance with energy dissipation. ACS Appl. Mater. Interfaces.

[49] Grayli S.V., Patel T., van Kasteren B., Kokilathasan S., Tekcan B., Alan Tam M.C., Losin W.F., Odinotski S., Tsen A.W., Wasilewski Z.R. (2025). Near-Unity Absorption in Semiconductor Metasurfaces Using Kerker Interference. Nano Lett..

