# Supplementary material for: Structural–Material Coupling Enabling Broadband Absorption for a Graphene Aerogel All-Medium Metamaterial Absorber
Source: Nanomaterials (Basel). 2025 Dec 22;16(1):18. doi: 10.3390/nano16010018 (PMC12787457; doi:10.3390/nano16010018)
Supplement: Supplementary file 1 [file nanomaterials-16-00018-s001.zip › nanomaterials-4050124-supplementary.pdf]

# Supporting Information

## Structural–Material Coupling Enabling Broadband Absorption for a Graphene Aerogel All-Medium Metamaterial Absorber

*Kemeng Yan[1], Yuhui Ren [1,†,a)], Jiaxuan Zhang[1], Man Song[2], Xuhui Du[2], Meijiao Lu[1], Dingfan Wu[1], Yiqing Li[1], Jiangni Yun [1]*

*[1]School of Electronic Information, Northwest University, Xi'an, 710127, China*

*[2]Xi'an HengDa Microwave Technology Development Co., Ltd, Xi'an, 710100, China*

In Figure S1(a), the SEM image shows that the graphene consists of thin, randomly stacked sheets with a crumpled and wrinkled morphology. The presence of numerous folds indicates the high flexibility of the graphene sheets and helps suppress severe restacking. This microstructure provides abundant interfaces and promotes multiple scattering of electromagnetic waves, which is favorable for interfacial polarization. X-ray photoelectron spectroscopy (XPS) was conducted to elucidate the surface chemistry of the graphene sample, as shown in Figure S1(b1–b3). The survey spectrum (Figure S1(b1)) shows distinct C 1s and O 1s peaks, confirming that carbon and oxygen are the major constituents and that oxygen-containing functional groups are retained. The high-resolution C 1s spectrum (Figure S1(b2)) can be deconvoluted into three main components: C–C/C=C, C–O, and O–C=O. The O 1s spectrum (Figure S1(b3)) reveals four contributions: C–O, O–C=O, –OH, and adsorbed $H_2O$.

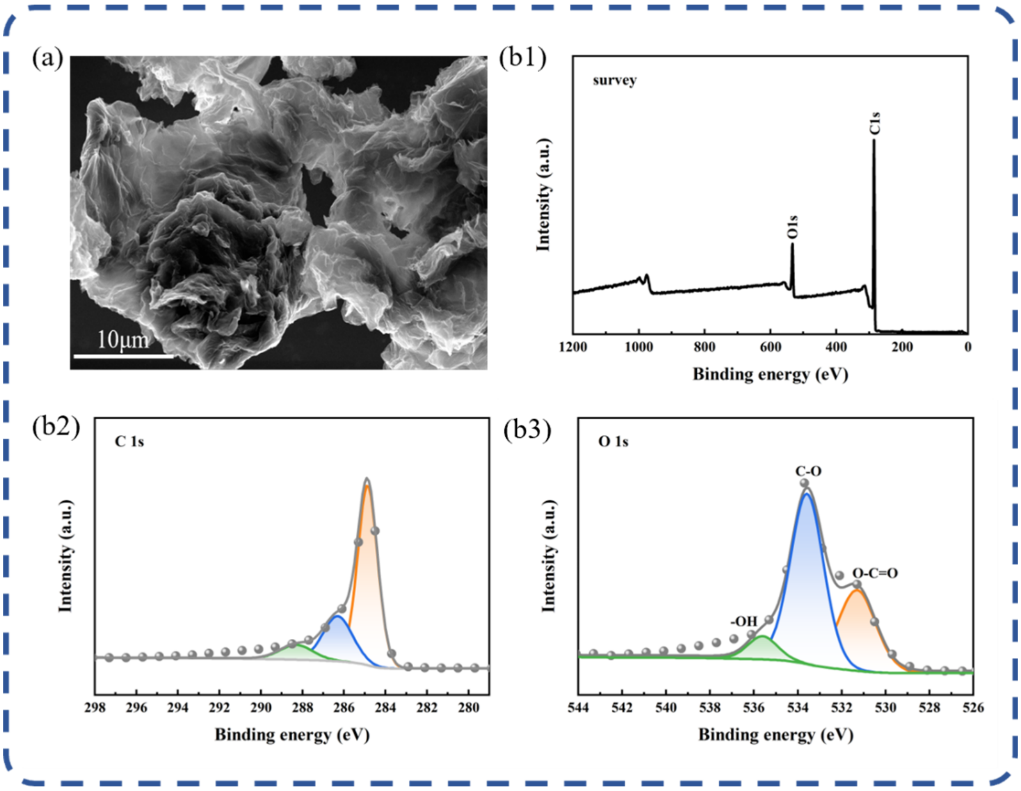


Figure S1. (a)SEM images of graphene; XPS analysis of graphene: (b1) survey spectrum; (b2) C 1s spectrum; and (b3) O 1s spectrum.

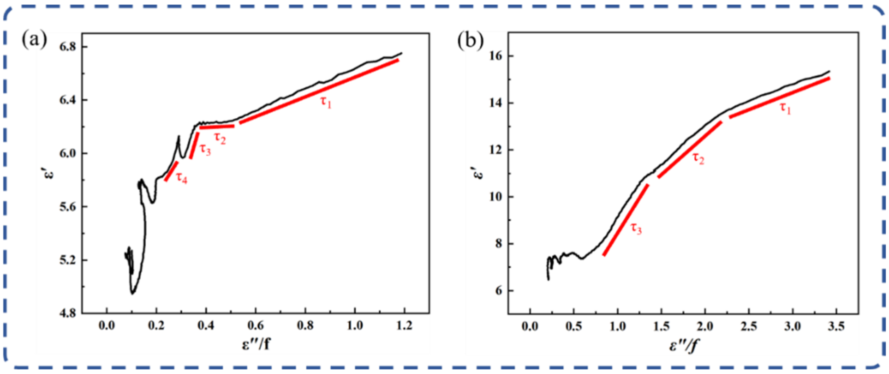


Figure S2. (a) The relationship between ε′ versus ε″/f of graphene; (b) The relationship between ε′ versus ε″/f of GA.

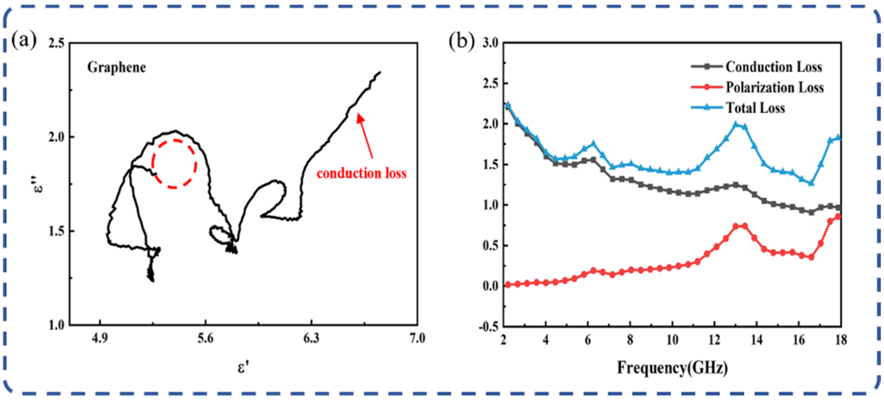


Figure S3. Graphene: (a) Cole–Cole plots; (b) Quantitively separation electrical losses.

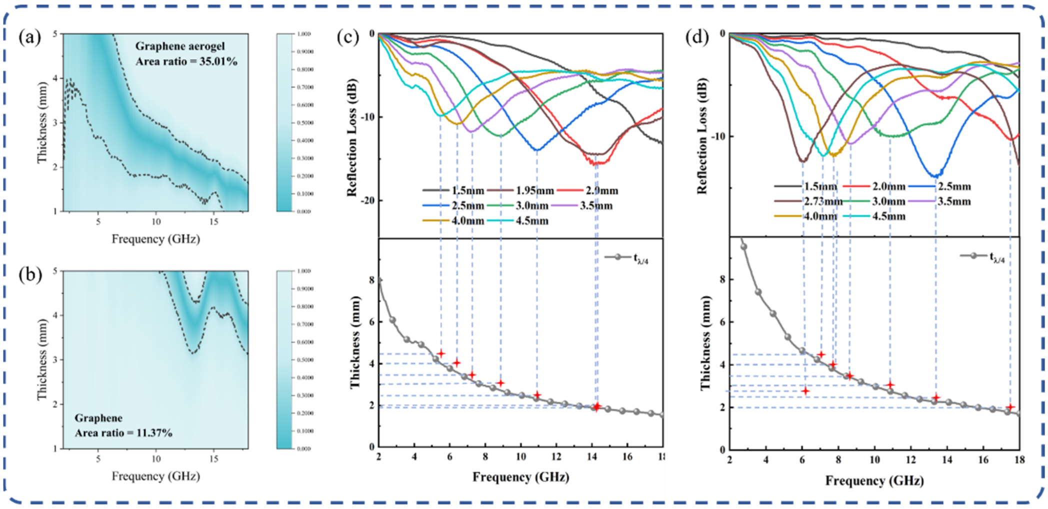


Figure S4. (a) The impedance matching of GA; (b) The impedance matching of graphene; (c) Quarter-wavelength thickness curves of GA; (d) Quarter-wavelength thickness curves of graphene.

**Table 1** Quantitively classified electrical losses

| Sample name | Maximum effective absorption bandwidth / GHz | Effective absorption frequency range / GHz | Average conductive loss within the band | Average relaxation polarization loss within the band |
|---|---|---|---|---|

| | | | | |
|---|---|---|---|---|
| Graphene | 2.83 | 11.3-14.1 | 69.2% | 30.8% |
| Graphene Aerogel | 6.46 | 11.5-18.0 | 28.2% | 71.8% |

**Details of Quantitative Electrical Loss Separation and Fitting Procedure**

This section provides additional methodological details on the quantitative separation of dielectric loss in graphene aerogel. The analysis is based on nonlinear least-squares fitting of the complex permittivity to distinguish conductive loss from relaxation polarization loss. These details are included to improve the transparency and reproducibility of the loss-separation procedure discussed in the main text. The fitting model, parameter constraints, boundary conditions, and quantitative error evaluation are described below.

(i) Fitting model, parameters, and constraints

The electrical loss separation is based on a nonlinear least-squares fitting of the complex permittivity using a multi-Debye relaxation model with an explicit conductive term, expressed as

$$\varepsilon^*(\omega) = \varepsilon_\infty + \sum_{i=1}^{N_r} \frac{\Delta\varepsilon_i}{1 + j\omega\tau_i} - j\frac{\sigma}{\omega\varepsilon_0},$$

where $\varepsilon_\infty$is the high-frequency limit of the permittivity, $\Delta\varepsilon_i$and $\tau_i$denote the dielectric strength and relaxation time of the $i$-th relaxation process, $\sigma$is the effective electrical conductivity, and $\varepsilon_0$is the vacuum permittivity.

Accordingly, the fitting parameter set is given by $\{\varepsilon_\infty, \sigma, \Delta\varepsilon_i, \tau_i\}, \quad i = 1, \ldots, N_r$.

To ensure physical consistency and numerical stability, several constraints were imposed during the nonlinear fitting process.

The electrical conductivity was restricted to $\sigma \geq 0$, reflecting the physical requirement that conductive loss cannot be negative. The relaxation times were constrained within the range of $10^{-13}\,\mathrm{s} \leq \tau_i \leq 10^{-6}\,\mathrm{s}$, which corresponds to typical time scales of dipolar and interfacial polarization in dielectric and microwave-absorbing materials. In addition, both $\varepsilon_\infty$and $\Delta\varepsilon_i$were restricted to positive values to maintain physically meaningful dielectric responses.

To further enhance numerical robustness, a segmented fitting strategy along the frequency axis was adopted. The entire frequency range was divided into several narrow sub-bands, within which both $\varepsilon'(\omega)$and $\varepsilon''(\omega)$were fitted simultaneously. The optimized parameters obtained from one sub-band were used as the initial values for the adjacent sub-band, ensuring smooth frequency evolution of the fitting parameters and avoiding nonphysical discontinuities.

(ii) Fitting uncertainty and error analysis

The fitting quality was not evaluated solely by visual agreement but was quantitatively assessed using frequency-dependent error metrics. Specifically, the relative error of the imaginary permittivity at each frequency point $\omega_k$was defined as

$$\delta_k = \frac{\left|\varepsilon''_{fit}(\omega_k) - \varepsilon_{exp''}(\omega_k)\right|}{\left|\varepsilon_{exp''}(\omega_k)\right| + \varepsilon_{tiny}},$$

where $\varepsilon''_{\mathrm{exp}}$and $\varepsilon''_{\mathrm{fit}}$represent the experimentally measured and fitted values, respectively,

and $\varepsilon_{\text{tiny}}$ is a small constant introduced to avoid numerical instability when the denominator approaches zero.

Based on this definition, two global error metrics were reported: the mean relative error

$$\overline{\delta} = \frac{1}{N}\sum_{k=1}^{N}\delta_k,$$

and the maximum relative error

$$\delta_{max} = max_k\ \delta_k.$$

For representative samples graphene and GA, the mean relative errors were 1.98% and 3.33%, respectively, while the maximum relative errors were 5.38% and 9.70%. These results indicate that the proposed model reproduces the experimental electrical loss behavior with good quantitative accuracy over the entire frequency range, supporting the reliability of the separation between conductive and polarization-related losses.

It should be emphasized that the objective of the fitting is not to obtain a mathematically unique set of relaxation parameters, but to achieve a physically constrained quantitative separation of conductive and polarization losses. The reported error metrics provide direct numerical evidence for the robustness and reliability of this loss-separation approach.
